# How have people been coping during the COVID-19 pandemic? Patterns and predictors of coping strategies amongst 26,016 UK adults

**DOI:** 10.1186/s40359-021-00603-9

**Published:** 2021-07-15

**Authors:** Meg Fluharty, Daisy Fancourt

**Affiliations:** Department of Behavioural Science and Health, University College London, 1-19 Torrington Place, London, WC1E 7HB UK

**Keywords:** COVID-19, Coping, Adversity

## Abstract

**Background:**

Individuals face increased psychological distress during the COVID-19 pandemic. However, it’s unknown whether choice of coping styles are influenced by COVID-19 in addition to known predictors.

**Methods:**

Data from 26,016 UK adults in the UCL COVID-19 Social Study were analysed from 12/4/2020 15/5/2020. Regression models were used to identify predictors of coping styles (problem-focused, emotion-focused, avoidant, and socially-supported): model 1 included sociodemographic variables, model 2 additionally included psychosocial factors, and model 3 further included experience of COVID-19 specific adverse worries or events.

**Results:**

Sociodemographic and psychosocial predictors of coping align with usual predictors of coping styles not occurring during a pandemic. However, even when controlling for the wide range of these previously known predictors specific adversities were associated with use of specific strategies. Experience of worries about finances, basic needs, and events related to Covid-19 were associated with a range of strategies, while experience of financial adversities was associated with problem-focused, emotion-focused and avoidant coping. There were no associations between coping styles and experiencing challenges in meeting basic needs, but Covid-19 related adversities were associated with a lower use of socially-supported coping.

**Conclusions:**

This paper demonstrates that there are not only demographic and social predictors of coping styles during the COVID-19 pandemic, but specific adversities are related to the ways that adults cope. Furthermore, this study identifies groups at risk of more avoidant coping mechanisms which may be targeted for supportive interventions.

**Supplementary Information:**

The online version contains supplementary material available at 10.1186/s40359-021-00603-9.

## Background

The coronavirus (COVID-19) pandemic has had diverse negative psychological effects globally. Individuals have experienced a wide range of adversities due to the virus, including those relating to the virus itself (such as experiencing illness oneself, concerns for friends and family, and bereavement), financial adversities (including loss of work or income, and inability to pay bills), and challenges in meeting basic needs (such as accessing sufficient food, medicine, and safe accommodation) [1]. Recent research has highlighted substantial increases in mental illness and loneliness during the COVID-19 pandemic [2, 3]. Whilst some of these experiences reflect those reported during previous pandemics [4], COVID-19 is causing greater concern due to the global scale, heavy lockdown measures implemented, and long time scale predicted [5].

As a large proportion of the global population has experienced some form of psychological distress during the pandemic, there have been calls for more research exploring factors that help to buffer against or exacerbate experiences [6]. This is particularly important given there are projected long-lasting effects of the COVID-19 pandemic alongside limited mental health resources available [7]. However, there were inequalities in those who were more likely to be negatively affected by pandemic-related stressors, with certain groups including younger adults, women, people from Black Asian Minority Ethic (BAME) groups, and people living alone experiencing poor mental health [4]. Differences in mental health responses are likely be influenced by differences in individuals’ use of various coping strategies. Therefore understanding coping strategies could help to identify the social and personal resources required by individuals to mitigate psychological stress as COVID-19 continues, and in future pandemics.

Coping is broadly defined as the conscious or unconscious cognitive and behavioural strategies an individual employs to manage stress [8, 9]. Numerous coping strategies have been identified, including self-distraction, active coping, denial, substance use, use of emotional support, use of informational support, and behavioural changes. These different coping strategies are often categorised into different groups. For example, 'approach' strategies typically focus on the stressor and one’s actions towards it (e.g. seeking emotional support or planning to resolve and reduce stressors)[10], while by contrast, 'avoidant' strategies seek to avoid the stressor and ones reaction to it (e.g. withdrawing from others, substance use, and denying the reality of the stressor) [11, 12]. Additional groupings of focus on whether activities are 'emotion-focused' (aiming to manage emotional distress; e.g. denial, venting, emotional support) or 'problem-focused' (efforts to modify the problem at hand; e.g. informational support, active coping) [13]. There is much debate as to whether certain strategies are more beneficial than others. For example, avoidance strategies may be helpful in reducing short term stress, but are generally considered harmful from the perspective of physical well-being as no direct actions are taken to reduce the stressor, leaving the individual to feel helpless or self-blaming [11, 14–17].

Previous studies have identified a range of predictors for coping style choice. Evidence suggests lower SEP is associated with greater use of avoidant strategies. These individuals have increased likelihood of exposure to stressors across the life course and may have less efficient coping strategies as a result of the social resources needed to combat stressors as well as less access to social support [18]. Personality type may influence coping strategy choice indirectly (influencing severity of stressors and effectiveness of coping) or directly by facilitating how individuals engage or disengage with threats and stressors (e.g. threat sensitivity in neurotic individuals may result in disengagement, while highly social extraverts may seek more supportive coping) [19, 20]. Furthermore, the way individuals react to stressors can have long term health effects [21, 22]. Avoidance strategies are typically at the core of depression and anxiety [23], which is why the most effective therapies (i.e. cognitive-behavioural therapy) focus on cognitive reappraisal and problem solving responses [24]. For example when faced with a traumatic event, adoption of avoidant strategies are associated with later mental health problems. This is particularly pertinent when considering individuals’ psychological responses to adversities during the COVID-19 pandemic as it is possible that coping strategies may be influenced solely by existing traits. However, it is also possible that the unusual and adverse circumstances of the pandemic may affect individuals’ coping resources and alter usual psychological responses [25]. It is vital to understand these patterns and predictors of coping strategies in order to identify who is most at need of additional psychological support.

Therefore, this study examined predictors of coping strategies amongst adults during the COVID-19 pandemic. Specifically, we explored (i) whether sociodemographic predictors of coping strategies align with usual predictors not during a pandemic, (ii) whether psychosocial factors including individuals’ roles during the pandemic, their living situation and their health status affected their use of coping strategies, and (iii) whether specific adverse experiences during the pandemic predisposed individuals to using more avoidant coping strategies above and beyond trait sociodemographic and psychosocial factors.

## Methods

### Participants

Data were drawn from the COVID-19 Social Study; a large panel study of the psychological and social experiences of over 70,000 adults (aged 18 +) in the United Kingdom (UK) during the COVID-19 pandemic. The study commenced on 21st March 2020 and involves online weekly data collection from participants for the duration of the COVID-19 pandemic in the UK. The study is not random and therefore is not representative of the UK population. But it does contain a well-stratified sample that was recruited using three primary approaches. First, snowballing was used, including promoting the study through existing networks and mailing lists (including large databases of adults who had previously consented to be involved in health research across the UK (e.g. UCL BioResource and Health Wise Wales), print and digital media coverage, and social media. Second, more targeted recruitment was undertaken focusing on (i) individuals from a low-income background (recruitment via via Find Out Now, SEO Works, FieldworkHub, and Optimal Worskhop, (ii) individuals with no or few educational qualifications, and (iii) individuals who were unemployed. Third, the study was promoted via partnerships with third sector organisations to vulnerable groups (e.g. UKRI MARCH Mental Health Research Network), including adults with pre-existing mental health conditions, older adults, carers, and people experiencing domestic violence or abuse.

Questions on coping were asked during a week-long module that was introduced in week 8 of the study 09th to 15th May. A total of 29,882 participants completed these questions in addition to all completing a detailed questionnaire on baseline sociodemographic factors and weekly data on experiences during COVID-19 during the period from 21st March until 15th May. Those who responded 'prefer not to say' to gender (0.43%) and income (9.4%) variables were set to missing, and we excluded participants who were missing data across any of the predictor variables (n = 3,302). An additional 390 participants were excluded as they did not have a baseline wave used to derive survey weights (although took part in the demographic part of the survey at later waves), which left a total complete case analytical sample size of 26,016 (Additional file 1: Table S1).

### Measures

#### Coping strategies

Coping was assessed by asking ‘how have you been coping during lockdown’ and measured using the 28-item brief-COPE questionnaire; a short version of the original 60-item scale. This scale determines primary coping styles as either approach or avoidant and covers the following domains of coping: self-distraction, active coping, denial, substance use, use of emotional support, use of instrumental support, behavioural disengagement, venting, positive reframing, planning, humour, acceptance, religion, & self-blame [26, 27]. In line with previous research, we used a previously-derived 4 factor model for our analyses: problem focused coping (active coping, planning), emotion focused coping (positive reframing, acceptance, humour, religion) avoidant coping (behavioural disengagement, denial, substance use), and socially supported coping (emotional support, instrumental support, and venting) [28].

#### Sociodemographic predictors

Six sociodemographic predictors were collected at baseline interview: (1) gender (male vs female), (2) age group were chosen to represent younger, middle, and older ages (18–29 vs 30–59 vs 60 +), (3) ethnicity (white vs BAME), (4) educational attainment (General Certificate of Secondary Education (GCSE) or lower (qualifications at age 16) vs A-Levels or vocational training (qualifications at age 18) vs undergraduate degree vs postgraduate degree), (5) low household income (< £30,000 per annum vs ≥ £30,000 per annum), and (6) employment status (employed vs student vs inactive vs unemployed).

#### Psychosocial predictors

Eight psychosocial predictors were collected at baseline interview: (1) area of dwelling (urban vs rural), (2) living status (alone vs not alone with children vs with others no children), (3) household overcrowding (alone vs with others-not overcrowded vs with others- overcrowded), (4) keyworker status was derived from responses to the question 'Are you currently fulfilling any of the government’s identified 'keyworker' roles?' (keyworker vs non-keyworker), (5) mental health condition (reports of a diagnosis vs none), (6) long term/pre-existing physical health condition or disability (reports of a diagnosis vs none), (7) number of close friends (continuous 1–10 +), (8) Social support was measured using an adapted version of the six-item short form of Perceived Social Support Questionnaire (F-SozU K-6) [29, 30]. Each item was rated on a 5-point scale from “not true at all” to “very true”, with higher scores indicating higher levels of perceived social support. Minor adaptations were made to the language in the scale to make it relevant to experiences during COVID-19 (see Additional file 1: Table S2 for a comparison of changes).

Two psychosocial predictors were asked as repeated questions each week and responses for this analysis were taken from week 8 of the study: [9] personality was measured using the Big Five Inventory (BFI-2), which measures five domains and 15 facets: Extraversion (sociability, assertiveness, and energy level), Agreeableness (compassion, respectfulness, and trust), Conscientiousness (organisation, productiveness, and responsibility), Nervousness (anxiety, depression, and emotional volatility), and Openness (intellectual curiosity, aesthetic sensitivity, and creative imagination) [31]. Each item uses a 5-point scale ranging from “strongly disagree” to “strongly agree”, with higher score indicating greater levels of each domain. Finally, [10] Loneliness was measured using the 3-item UCLA-3 loneliness, a short form of the Revised UCLA Loneliness Scale (UCLA-R) [32]. Each item was rated with a 4-point rating scale, ranging from “never” to “always”, with higher score indicating greater loneliness, scores were averaged across each week.

#### Adversity predictors

Repeated questions each week assessed participants’ experience of adversities and responses for this analysis were taken from week 8 of the study: (1) Covid-19 status (positive/suspected vs none); (2) experience of one of a number of specific adversities including financial adversities (yes to any of the following: whether participants had lost their job or been unable to work, their partner had lost their job or was unable to work, or they had experienced a major cut in household income), challenges meeting basic needs (yes to any of the following: whether participants had lost their accommodation, they had been unable to access sufficient food, or they had been unable to access required medication), and virus related adversities (yes to any of the following: whether in the past week the participant had suspected or diagnosed COVID-19, somebody close to them was hospitalised, or they had lost somebody close to them) [33]; and (3) adversity worries were captured from two questions that asked participants to select which of a list of items had caused them major stress in the past week financial stressors (yes to any of the following: losing your job/unemployment), stressors relating to meeting basic needs (yes to any of the following: your own safety/security, getting food, and getting medication), and stressors relating to the virus (catching or becoming seriously ill from COVID-19) [34].

### Analysis

We used fixed-effects ordinary least squares regression models to identify predictors of coping styles. 3 additive models were applied to each of the coping styles in a forward stepwise selection. Model 1 included sociodemographic variables, Model 2 additionally included psychosocial factors (Model 2), and Model 3 additionally included experience of specific adverse worries or events.

Complete model specifications are as follows:

- *Model 1*: [coping style ~ sociodemographic predictors]
- *Model 2*: [coping style ~ sociodemographic + psychosocial predictors]
- *Model 3*: [coping style ~ gender + sociodemographic + psychosocial + adversity predictors]

To account for the non-random nature of the sample, data were weighted to the proportions of age group, gender and educational level on the basis of Office for National Statistics (ONS) population estimates [35]. A cross-sectional weight variable was created for all participants at baseline using the Stata user-written command ‘ebalence’ [36]. All analyses were carried out in Stata version 16.0 (Statacorps, Texas).

## Results

Characteristics of the study sample (both unweighted and weighted samples) are shown in Table 1. 51% of participants in the weighted sample were female, 91% were of white ethnicity, 45% aged 30–49, and 60% were in full time employment.

**Table 1:** Sample demographics (%), weighted and unweighted figures

	Unweighted data (%)	Weighted data (%)
Gender		
Male	25.2	49.3
Female	74.8	50.7
Age group		
18–29	6.5	10.4
30–59	58.3	47.9
60 +	35.2	41.7
Ethnicity		
White	96.1	91.7
BAME	3.9	8.4
Education		
GCSE or Lower	12.7	30.2
A-Level or Vocational	16.7	32.5
Undergraduate degree	41.8	22.2
Postgraduate degree	28.8	15.2
Employment		
Employed	62.2	54.3
School	2.7	4.5
Inactive	33.3	38.9
Unemployed	1.8	2.3
Ownership		
Owned	75.9	70.0
Rented/other	24.2	30.1
Low income		
No	61.4	51.9
Yes	38.6	48.1
Area		
Urban	76	77.2
Rural	24.3	22.8

The use of problem-focused coping in the sample range from − 0.75 to 1.38 (M =:  0.01, SD = 0.50) with skewness of 0.25 and kurtosis of 2.57, Use of emotion-focused coping ranged from − 1.45 to 1.59 (M = 0.00, SD = 0.66) with skewness of 0.14 and kurtosis of 2.77. Use of avoidant coping ranged from -0.45 to 1.48 (M = 0.08, SD = 0.53) with skewness of 0.98 and kurtosis of 3.20, while use of socially supported coping ranged from -0.91 to 1.71 (M = -0.01, SD = 0.67) with skewness of 0.37 and kurtosis of 2.53.

### Sociodemographic predictors

Women were more likely to use all coping strategies than men (Table 2 [Model 1]). Older adults were less likely to use avoidant and socially supported coping strategies. There were no associations observed for coping strategy by ethnicity. Higher educational attainment was associated with more use of problem-focused, emotion focused, and socially supportive strategies. People who were ‘inactive’ in terms of employment (i.e. retired or home-makers) were less likely to use problem or emotion-focused coping. In terms of SEP, lower SEP (indicated by not owning a home and having lower household income) was associated with greater use of disengagement strategies, while low income was also associated with less use of active and supportive strategies.

**Table 2:** Associations of sociodemographic factors with coping styles

	Coping 1: Problem-focused				Coping 2: Emotion-focused				Coping 3: Avoidant				Coping 4: Socially supported			
	Coef	95% CI		*P*	Coef	95% CI		*P*	Coef	95% CI		*P*	Coef	95% CI		*P*
Gender																
Male	*				*				*				*			
Female	0.10	0.09	0.12	< 0.001	0.21	0.18	0.23	< 0.001	0.08	0.06	0.11	< 0.001	0.27	0.25	0.30	< 0.001
Age group																
18–29	*				*				*				*			
30–59	0.04	0.00	0.08	0.056	0.00	− 0.06	0.05	0.928	− 0.07	− 0.12	− 0.03	0.003	− 0.21	− 0.26	− 0.16	< 0.001
60 +	0.03	− 0.02	0.07	0.295	0.02	− 0.04	0.08	0.551	− 0.18	− 0.23	− 0.12	< 0.001	− 0.32	− 0.38	− 0.26	< 0.001
Ethnicity																
White	*				*				*				*			
BAME	0.03	− 0.02	0.08	0.236	0.06	0.00	0.13	0.067	− 0.02	− 0.07	0.03	0.363	− 0.06	− 0.12	0.00	0.056
Education																
Highest qual/GCSE lower	*				*				*				*			
A levels or vocational training	0.06	0.04	0.09	< 0.001	0.10	0.06	0.14	< 0.001	− 0.01	− 0.04	0.02	0.374	0.09	0.05	0.12	< 0.001
Undergraduate degree	0.13	0.11	0.16	< 0.001	0.17	0.13	0.20	< 0.001	− 0.02	− 0.05	0.00	0.067	0.22	0.19	0.26	< 0.001
Postgraduate degree	0.21	0.18	0.24	< 0.001	0.21	0.18	0.25	< 0.001	− 0.04	− 0.07	− 0.01	0.004	0.35	0.32	0.38	< 0.001
Employment																
Employed	*				*				*				*			
Student	0.08	0.02	0.13	0.010	0.05	− 0.03	0.13	0.198	0.08	0.00	0.16	0.045	0.08	0.00	0.17	0.062
Inactive	− 0.05	− 0.08	− 0.02	< 0.001	− 0.06	− 0.10	− 0.03	< 0.001	0.03	0.00	0.05	0.074	0.01	− 0.03	0.04	0.719
Unemployed	0.07	− 0.01	0.14	0.068	− 0.03	− 0.12	0.05	0.434	0.01	− 0.07	0.09	0.762	− 0.06	− 0.13	0.02	0.154
Ownership																
Owned	*				*				*				*			
Rented/other	0.00	− 0.02	0.02	0.844	0.01	− 0.02	0.04	0.415	0.09	0.06	0.11	< 0.001	0.05	0.02	0.08	0.001
Low income																
No	*				*				*				*			
Yes	− 0.01	− 0.03	0.01	0.464	− 0.05	− 0.08	− 0.02	0.001	0.05	0.02	0.07	< 0.001	− 0.07	− 0.10	− 0.04	< 0.001

Displays Model 1 predictors; star indicates reference group

Fit statistics: problem focused: R^2^ = 0.042, AIC = 1.397; emotion-focused: R^2^ = 0.046, AIC = 1.921; avoidant: R^2^ = 0.027, AIC = 1.487; socially supported: R^2^ = 0.104, AIC = 1.941

### Psychosocial predictors

Even when controlling for sociodemographic predictors, individuals living in over-crowded households were more likely to use avoidant strategies, whilst individuals living alone were more likely to use a range of coping strategies (Table 3 [Model 2]; full results available in Additional file 1: Table S3). Individuals living in rural areas were less likely to draw on avoidant or support strategies. Individuals who were lonely were more likely to use a rage of coping strategies, as were those with social support although this was protective against avoidant coping. Keyworkers were less likely to use problem-focused or emotion-focused coping strategies. People with a diagnosed mental health condition were more likely to use avoidant coping and supportive coping, while those with a health condition used supporting strategies. All personality types were generally associated with greater use of all strategies, with the exception, and conscientiousness being associated with lower levels of support, and avoidant, strategies.

**Table 3:** Associations of psychosocial characteristics with coping styles

	Coping 1: Problem-focused				Coping 2: Emotion-focused				Coping 3: Avoidant				Coping 4: Socially supported			
	Coef	95% CI		*P*	Coef	95% CI		*P*	Coef	95% CI		*P*	Coef	95% CI		*P*
Area																
Urban	*				*				*				*			
Rural	0.00	− 0.02	0.03	0.640	0.00	− 0.02	0.03	0.735	− 0.03	− 0.05	− 0.01	0.005	− 0.03	− 0.06	− 0.01	0.009
Overcrowding																
Living w/ others not crowded	*				*				*				*			
Alone	0.01	− 0.02	0.03	0.646	0.04	0.00	0.07	0.033	− 0.09	− 0.12	− 0.07	< 0.001	0.06	0.03	0.08	< 0.001
Living w/ others overcrowded	0.03	0.00	0.07	0.041	0.05	0.00	0.09	0.039	0.04	0.01	0.08	0.021	0.05	0.01	0.09	0.024
Keyworker																
No	*				*				*				*			
Yes	− 0.03	− 0.06	− 0.01	0.003	− 0.05	− 0.08	− 0.01	0.006	0.01	− 0.01	0.04	0.319	− 0.01	− 0.03	0.02	0.731
Diagnosed physical condition																
None	*				*				*				*			
Yes	− 0.01	− 0.03	0.01	0.488	− 0.01	− 0.04	0.01	0.273	0.00	− 0.02	0.02	0.713	0.03	0.00	0.05	0.025
Diagnosed mental condition																
None	*				*				*				*			
Yes	0.00	− 0.03	0.02	0.809	0.02	− 0.01	0.06	0.209	0.10	0.08	0.13	< 0.001	0.08	0.05	0.11	< 0.001
Personality																
Neuroticism	0.01	0.01	0.01	< 0.001	0.00	0.00	0.00	0.951	0.02	0.02	0.02	< 0.001	0.03	0.03	0.03	< 0.001
Extraversion	0.01	0.01	0.01	< 0.001	0.01	0.01	0.01	< 0.001	0.01	0.01	0.01	< 0.001	0.01	0.01	0.01	< 0.001
Openness	0.03	0.02	0.03	< 0.001	0.03	0.03	0.03	< 0.001	0.00	0.00	0.01	0.072	0.02	0.02	0.02	< 0.001
Agreeableness	0.00	0.00	0.01	0.137	0.01	0.01	0.02	< 0.001	0.00	− 0.01	0.00	0.021	0.00	0.00	0.00	0.461
Conscientiousness	0.01	0.01	0.02	< 0.001	0.00	0.00	0.00	0.914	− 0.01	− 0.01	0.00	< 0.001	− 0.01	− 0.01	− 0.01	< 0.001
Loneliness																
Average loneliness score	0.04	0.03	0.04	< 0.001	0.02	0.01	0.02	< 0.001	0.09	0.08	0.09	< 0.001	0.06	0.06	0.07	< 0.001
Social support																
Social support score	0.01	0.01	0.01	< 0.001	0.02	0.02	0.02	< 0.001	− 0.01	− 0.01	0.00	< 0.001	0.04	0.04	0.04	< 0.001

Displays Model 2 predictors; star indicates reference group

Fit statistics: problem focused: R^2^ = 0.118, AIC = 1.316; emotion-focused: R^2^ = 0.131, AIC = 1.829; avoidant: R^2^ = 0.200, AIC = 1.292; socially supported: R^2^ = 0.291, AIC = 1.708

### Specific events and worries

Even when controlling for the wide range of sociodemographic and psychosocial factors in models 1 and 2, specific adversities were associated with use of specific strategies (Table 4 [Model 3]; full results available in Additional file 1: Table S4). Experience of worries about finances, basic needs, and events related to Covid-19 were associated with a range of strategies, while experience of financial adversities was associated with problem-focused, emotion-focused and avoidant coping. There were no associations between coping styles and experiencing challenges in meeting basic needs, but Covid-19 related adversities were associated with a lower use of socially-supported coping.

**Table 4:** Associations of worries and adverse events with coping styles

	Coping 1: Problem-focused				Coping 2: Emotion-focused				Coping 3: Avoidant				Coping 4: Socially supported			
	Coef	95% CI		*P*	Coef	95% CI		*P*	Coef	95% CI		*P*	Coef	95% CI		*P*
Ever had Covid-19																
No	*				*				*				*			
Yes	0.04	0.01	0.07	0.006	0.06	0.02	0.09	0.003	0.03	0.00	0.06	0.058	0.07	0.03	0.10	< 0.001
Adverse events: finances																
No	*				*				*				*			
Yes	0.08	0.05	0.10	< 0.001	0.04	0.01	0.08	0.015	0.07	0.04	0.10	< 0.001	0.01	− 0.03	0.04	0.717
Adverse events: basic needs																
No	*				*				*				*			
Yes	0.02	− 0.04	0.09	0.495	0.00	− 0.10	0.09	0.951	0.06	− 0.03	0.15	0.167	0.02	− 0.07	0.10	0.677
Adverse events: Covid− 19																
No	*				*				*				*			
Yes	− 0.06	− 0.16	0.03	0.205	− 0.07	− 0.21	0.06	0.298	0.05	− 0.07	0.18	0.387	− 0.15	− 0.26	− 0.04	0.008
Worries: finances																
No	*				*				*				*			
Yes	0.09	0.07	0.11	< 0.001	0.04	0.01	0.07	0.005	0.08	0.06	0.10	< 0.001	0.10	0.07	0.13	< 0.001
Worries: basic needs																
No	*				*				*				*			
Yes	0.05	0.03	0.08	< 0.001	0.02	− 0.01	0.05	0.255	0.06	0.04	0.09	< 0.001	0.11	0.08	0.14	< 0.001
Worries Covid-19																
No	*				*				*				*			
Yes	0.11	0.09	0.13	< 0.001	0.09	0.07	0.12	< 0.001	0.02	0.00	0.04	0.027	0.08	0.05	0.10	< 0.001

Displays Model 3 predictors; star indicates reference group

Fit statistics: problem focused: R^2^ = 0.142, AIC = 1.289; emotion-focused: R^2^ = 0.135, AIC = 1.826; avoidant: R^2^ = 0.215, AIC = 1.275; socially supported: R^2^ = 0.309, AIC = 1.683

Unweighted analyses for all models are provided in Additional file 1: Tables S5–S7.

## Discussion

This study explored predictors of coping strategies during the COVID-19 pandemic. Active coping strategies were more common amongst women, older adults, people with higher educational attainment, people who were employed, people with higher income, but was less strongly predicted by psychosocial factors.

Problem-focused and emotion-focused coping strategies were more common amongst women, people with higher educational attainment, and those in school, but less strongly predicted by psychosocial factors. Supportive coping strategies were similarly more common in women, and people with higher education but also amongst younger adults and people with higher income. People living with others were more likely to draw on support strategies, as were people who lonely, who lived in urban areas, and who had a diagnosed mental health condition. Avoidant coping strategies were used more by women, younger adults, and people of lower educational attainment and lower socio-economic position, as well as people living with others, and people with mental health conditions and people who were more lonely.

The demographic predictors of coping including gender [37, 38] and age [39, 40], and age align with usual predictors of coping styles not occurring during a pandemic. For example, women scored higher on a range of coping styles compared to men [37]. Older adults were less likely to use lower engagement avoidant and socially supported strategies, which may result from accumulated experience with stressors leading to the adoption of more pro-active approaches [39, 40]. Further, there were no apparent differences in coping styles depending on ethnicity. This slightly contrasts with previous studies, which have suggested that individuals from BAME groups are more likely to use alternative coping styles such as religion [38, 41, 42]. But as religion was incorporated within emotion focused coping, this finding may have been obscured. Socioeconomic predictors of coping strategies echoed previous studies, with disadvantaged groups more likely to use avoidant coping strategies [18, 43]. Therefore, this study found that the usual demographic predictors of coping strategies were preserved during the COVID-19 pandemic, suggesting that individual’s traits and socio-economic circumstances are at least partly responsible for differences in management of stressors during the pandemic.

However, over and above trait demographic factors, a number of psychosocial factors were also found to influence use of coping strategies. Diagnosed mental health conditions were associated with a heightened use of avoidant coping strategies, echoing previous studies [22, 23, 25]. Although, we also found evidence that people with depression and anxiety turned to supportive coping during the pandemic. This could have been as a direct result of schemes such as Mutual Aid groups, which explicitly tried to support individuals with mental health problems, and a heightened awareness of supporting mental wellbeing during the pandemic. Our findings that keyworkers made less use of problem and emotion-focused coping strategies go against previous research, which suggests that workers in areas such as nursing employ active coping to maintain psychological health and resilience [44, 45] However, one potential explanation for this divergence is that a number of keyworkers in the current study unexpectedly found themselves in critical roles (e.g. supermarket employees, delivery and transportation drivers) and lacked previous training or experience in developing specific supportive emotion regulation responses unlike medical professionals, who have been the focus of much of the previous research on coping strategies [46, 47]. Our research mirrors previous work showing that overcrowded living is associated with increased avoidant coping strategies [48]. Additionally, one study of living alone during the pandemic found an increase in substance use coping, although this study did not examine other methods beyond substance use [49]. With regards to loneliness, increased loneliness in our study was associated with a range of coping styles, which is also supported by previous evidence [50]. But social support as a coping predictor outside clinical populations [51, 52] has not typically been examined. Here we found it associated with decreased avoidant coping, but further research is needed to understand whether this relationship is an artefact of the COVID-19 context or a more general indicator of social predictors of coping styles.

What is most notable, however, is that certain specific events related to the Covid-19 pandemic were also associated with the use of different coping strategies, even after adjusting for sociodemographic factors, and psychosocial characteristics. The finding that events involving Covid-19 adversities were associated with less socially supported strategies, while worries about these events were associated with a range of coping strategies. This suggests that individuals’ have a more positive outlook of how they envision handling certain situations versus the trauma of actually experiencing them. This is supported by previous research showing that people respond more positively in their coping styles to hypothetical situations than to situations for which they have prior experience (such as bullying). The decreased probability of using socially supported coping strategies could underlie the relationship being reported between worries and adversities relating to the virus during the pandemic and poorer levels of depression and anxiety shown in other research [34]. This is concerning because coping styles aimed at addressing the problems directly have been associated with positive affect and less association with negative affect, while avoidance styles display the opposite [53]. Coping styles are thought to initiate, modulate, and maintain affective responses, therefore avoidance coping is the least beneficial as it blocks attempts to address the stressors/problem and further blocks awareness that the situation may change. While this can be an effective short term strategy for distracting and resting from a stressor, prolonged reliance on avoidance coping may be harmful as the situation is not changed and individuals are engaging with the stressor for prolonged periods which in turn maintains negative affect [53–55].

This paper demonstrates that there are not only demographic and social predictors of coping styles during the COVID-19 pandemic, but specific adversities are related to the ways that adults cope. Whilst there are some concerning patterns suggesting that certain groups are at greater risk of using avoidant coping strategies, there is also evidence that individuals can change their coping strategies over time. So coping could be a target for interventions designed to improve mental health during the pandemic. Two approaches could be considered here. First, whilst changing demographic predictors is not a feasible intervention, it is possible that interventions targeting psychosocial factors or specific adversities could provide support. For example, supporting individuals in developing their social networks has been shown to help individuals engage with positive coping during the pandemic [56]. Second, previous studies have shown that techniques such as Cognitive Behavioural Therapy, stress management apps, and seeking social support can be used to increase adaptive coping strategies [23, 57, 58]. This shift has been found not just to change in-the-moment coping, but also to enhance psychosocial outcomes. For example, lonely individuals who learn more active coping strategies are able to reduce their loneliness [50]. Similarly, people with mental health problems have been found to experience a reduction in negative symptoms when shifting from avoidant to adaptive coping through the use of cognitive behavioural therapies [24]. This has been shown specifically for people in isolation too: improvements in mental health have been found for people in prison if they manage to adopt new coping strategies [59, 60]. Given the evidence in this study of clear socio-demographic predictors of coping strategies, such interventions could be specifically targeted at individuals in more deprived areas, and those experiencing financial loss [61].

This study has a number of strengths including its large sample size, its longitudinal tracking of participants used to identify adversities and worries across the first 8 weeks of lockdown, and its rich inclusion of measures on psychological and social experiences during COVID-19. We measured coping using the brief-COPE, a large validated measure. Further, a large portion coping literature is centred around specific traumatic events (e.g. health diagnosis, war, or abuse) and therefore it’s difficult to determine general population versus specific event predictors. However, in our three models we separated out known trait predictors from COVID-19 specific predictors. However, there are several limitations. The study is not nationally representative, although it does have good stratification across all major socio-demographic groups and analyses were weighted on the basis of population estimates of core demographics. Whilst the recruitment strategy deliberately over-sampled from groups such as individuals those from a low-income background, individuals with no or few educational qualifications, and individuals who were unemployed, it is possible that more extreme experiences were not adequately captured. Coping was only measured at one timepoint and therefore, we were not able to examine changes in coping strategy across time. Furthermore, it is possible that individuals experiencing highest levels of adversities including bereavement during the pandemic may have dropped out prior to week eight when the measures on coping were asked, or the sampling may have been selective towards individuals more likely to engage with positive coping strategies as undertaking a weekly questionnaire was arguably an approach-focused strategy. Nevertheless, we had good spread across possible responses for each of the measures included in the coping questionnaire and the sample remained heterogeneous.

Overall our study shows that a combination of trait demographic factors, psychosocial factors, and factors specific to experiences during the first UK lockdown in the COVID-19 pandemic predicted coping strategies. People most at risk of using avoidant coping strategies included those of lower socioeconomic position, with mental health conditions, higher rates of loneliness, and those experiencing COVID-19 related adverse events relating to finances and basic needs. This is noteworthy as the same groups have been identified as having poorer mental health experiences across this period, suggesting that one’s coping strategies could play an important role in how effectively individuals manage to cognitively and behaviourally manage stress during pandemics. It also highlights the importance of both providing specific support that will reduce individuals’ use of avoidant coping strategies such as digital or mutual aid [56, 62], and supporting and educating individuals (in particular those most at risk of adverse mental health outcomes) in how to use supportive coping strategies. Such work will be important as the COVID-19 pandemic continues and in the future to help mitigate the adverse psychological effects of such events.

## Supplementary Information

**Additional file 1.** Provides demographic characterics (Table S1); comparison of origional and revised F-SozU K-6 Questionnaire (Table S2); full model results (Tables S3 & S4), and unweighted models (Tabled S5–S7).

## Data Availability

The data was taken from the Covid-19 Social Study (www.covidsocialstudy.org), which is not currently open access (May 2021) but will be made available on a third-party archive following the end of the pandemic. Analytical code is available on Github: https://github.com/UCL-BSH/coping-predictors.

## Abbreviations

COVID-19: Coronavirus
BAME: Black Asian Minority Ethic
SEP: Socioeconomic position
UK: United Kingdom
GCSE: General Certificate of Secondary Education
F-SozU K-6: Perceived Social Support Questionnaire
BFI-2: Big Five Inventory
ONS: Office for National Statistics
