# Supplementary material for: How have people been coping during the COVID-19 pandemic? Patterns and predictors of coping strategies amongst 26,016 UK adults
Source: BMC Psychol. 2021 Jul 15;9:107. doi: 10.1186/s40359-021-00603-9 (PMC8280648; doi:10.1186/s40359-021-00603-9)
Supplement: Supplementary file 1 — Additional file 1. Provides demographic characterics (Table S1); comparison of origional and revised F-SozU K-6 Questionnaire (Table S2); full model results (Tables S3 & S4), and unweighted models (Tabled S5–S7). [file 40359_2021_603_MOESM1_ESM.docx]

Supplementary Table S1 Demographic characteristics (N=26,016)

| Sociodemographic | |  | Psychosocial | |  | Adverse events | |
| --- | --- | --- | --- | --- | --- | --- | --- |
|  | *%* |  |  | *%* |  |  | *%* |
| **Gender** |  |  | **Area** |  |  | **Ever had Covid-19** |  |
| Male | 49.3 |  | Urban | 77.2 |  | No | 0.891 |
| Female | 50.7 |  | Rural | 22.8 |  | Yes | 0.109 |
| **Age group** |  |  | **Overcrowding** |  |  | **Adverse events: finances** |  |
| 18-29 | 10.4 |  | Living alone | 0.6826 |  | No | 0.84 |
| 30-59 | 47.9 |  | Living w/ others not crowded | 0.2139 |  | Yes | 0.16 |
| 60+ | 41.7 |  | Living w/ others overcrowded | 0.1034 |  | **Adverse events: basic needs** |  |
| **Ethnicity** |  |  | **Keyworker** |  |  | No | 0.961 |
| White | 91.7 |  | No | 0.8049 |  | Yes | 0.039 |
| BAME | 8.4 |  | Yes | 0.1951 |  | **Adverse events: Covid-19** |  |
| **Education** |  |  | **Physical health condition** |  |  | No | 0.977 |
| Highest qual/GCSE lower | 30.2 |  | No | 0.5543 |  | Yes | 0.023 |
| A levels or vocational training | 32.5 |  | Yes | 0.4457 |  | **Worries: finances** |  |
| Undergraduate degree | 22.2 |  | **Mental health condition** |  |  | No | 0.535 |
| Postgraduate degree | 15.2 |  | No | 0.8253 |  | Yes | 0.465 |
| **Household income<30k** |  |  | Yes | 0.1747 |  | **Worries: basic needs** |  |
| No | 70 |  |  | *M (SD)* |  | No | 0.761 |
| Yes | 30.1 |  | **Big five: Neuroticism** | 11.07 (0.04) |  | Yes | 0.24 |
| **Employed** |  |  | **Big five: Extraversion** | 12.60 (0.04) |  | **Worries: Covid-19** |  |
| Employed | 4.5 |  | **Big five: Openness** | 14.94 (0.03) |  | No | 0.519 |
| School | 38.9 |  | **Big five: Agreeableness** | 15.37 (0.03) |  | Yes | 0.481 |
| Inactive | 2.3 |  | **Big five: Conscientiousness** | 15.70 (0.03) |  |  |  |
| Unemployed | 70 |  | **Loneliness** | 4.90 (0.02) |  |  |  |
|  |  |  | **Social support** | 21.03 (0.07) |  |  |  |

*Participants were excluded who did not list their gender as male/female, whom had missing data in any predictor variable, and missing weights*

Supplementary Table S2: Comparison of items in the original and revised Perceived Social Support Questionnaire (F-SozU K-6).

| Original | Adapted for COVID-19  In the past week, I feel… |
| --- | --- |
| I experience a lot of understanding and security from others | I have experienced a lot of understanding and support from others |
| I know a very close person whose help I can always count on | I have a very close person whose help I can always count on |
| If necessary, I can easily borrow something I might need from neighbours or friends | If necessary, I can easily borrow something I need from neighbours or friends |
| I know several people with whom I like to do things | I have people with whom I can spend time and do things together |
| When I am sick, I can without hesitation ask friends and family to take care of  important matters for me | If I get sick, I have friends and family who will take care of me |
| If I am down, I know to whom I can go without hesitation | If I am feeling down, I have people I can talk to without hesitation |

Table S3 Model 2: Associations of sociodemographic factors and psychosocial characteristics with coping styles

|  |  |  | **Coping 1: Problem-focused** | | | |  | **Coping 2: Emotion-focused** | | | |  | **Coping 3: Avoidant** | | | |  | **Coping 4: Socially supported** | | | |
| --- | --- | --- | --- | --- | --- | --- | --- | --- | --- | --- | --- | --- | --- | --- | --- | --- | --- | --- | --- | --- | --- |
|  |  |  | *Coef* | *95% CI* | | *P* |  | *Coef* | *95% CI* | | *P* |  | *Coef* | *95% CI* | | *P* |  | *Coef* | *95% CI* | | *P* |
|  | **Gender** | Male | * |  |  |  |  | * |  |  |  |  | * |  |  |  |  | * |  |  |  |
|  |  | Female | 0.04 | 0.03 | 0.06 | <0.001 | | 0.15 | 0.13 | 0.18 | <0.001 | | 0.03 | 0.01 | 0.05 | 0.002 |  | 0.15 | 0.13 | 0.17 | <0.001 |
|  | **Age group** | 18-29 | * |  |  |  |  | * |  |  |  |  | * |  |  |  |  | * |  |  |  |
|  |  | 30-59 | 0.07 | 0.03 | 0.11 | <0.001 | | 0.04 | -0.01 | 0.09 | 0.160 |  | -0.01 | -0.05 | 0.03 | 0.613 |  | -0.08 | -0.13 | -0.04 | <0.001 |
|  |  | 60+ | 0.07 | 0.03 | 0.12 | 0.002 |  | 0.04 | -0.02 | 0.10 | 0.146 |  | 0.00 | -0.05 | 0.05 | 0.934 |  | -0.10 | -0.16 | -0.05 | <0.001 |
|  | **Ethnicity** | White | * |  |  |  |  | * |  |  |  |  | * |  |  |  |  | * |  |  |  |
|  |  | BAME | 0.03 | -0.01 | 0.08 | 0.174 |  | 0.06 | 0.00 | 0.13 | 0.046 |  | -0.02 | -0.06 | 0.03 | 0.407 |  | -0.04 | -0.09 | 0.02 | 0.177 |
|  | **Education** | Highest qual/GCSE lower | * |  |  |  |  | * |  |  |  |  | * |  |  |  |  | * |  |  |  |
|  |  | A levels or vocational training | 0.04 | 0.02 | 0.07 | 0.001 |  | 0.08 | 0.05 | 0.12 | <0.001 | | -0.02 | -0.05 | 0.00 | 0.071 |  | 0.07 | 0.04 | 0.10 | <0.001 |
|  |  | Undergraduate degree | 0.10 | 0.07 | 0.12 | <0.001 | | 0.12 | 0.08 | 0.15 | <0.001 | | -0.02 | -0.04 | 0.01 | 0.200 |  | 0.20 | 0.17 | 0.23 | <0.001 |
|  |  | Postgraduate degree | 0.15 | 0.12 | 0.18 | <0.001 | | 0.14 | 0.10 | 0.17 | <0.001 | | -0.03 | -0.05 | 0.00 | 0.052 |  | 0.30 | 0.27 | 0.34 | <0.001 |
|  | **Employment** | Employed | * |  |  |  |  | * |  |  |  |  | * |  |  |  |  | * |  |  |  |
|  |  | Student | 0.02 | -0.03 | 0.08 | 0.374 |  | 0.00 | -0.07 | 0.08 | 0.907 |  | 0.01 | -0.06 | 0.08 | 0.843 |  | 0.01 | -0.07 | 0.08 | 0.832 |
|  |  | Inactive | -0.05 | -0.08 | -0.02 | <0.001 | | -0.07 | -0.10 | -0.03 | <0.001 | | -0.01 | -0.03 | 0.02 | 0.686 |  | -0.02 | -0.06 | 0.01 | 0.124 |
|  |  | Unemployed | 0.06 | -0.01 | 0.13 | 0.093 |  | -0.02 | -0.10 | 0.06 | 0.596 |  | -0.05 | -0.12 | 0.03 | 0.219 |  | -0.03 | -0.09 | 0.03 | 0.372 |
|  | **Ownership** | Owned | * |  |  |  |  | * |  |  |  |  | * |  |  |  |  | * |  |  |  |
|  |  | Rented/other | -0.02 | -0.04 | 0.01 | 0.180 |  | 0.03 | 0.00 | 0.06 | 0.098 |  | 0.01 | -0.02 | 0.03 | 0.533 |  | 0.05 | 0.02 | 0.08 | <0.001 |
|  | **Low income** | No | * |  |  |  |  | * |  |  |  |  | * |  |  |  |  | * |  |  |  |
|  |  | Yes | 0.01 | -0.02 | 0.03 | 0.640 |  | 0.00 | -0.03 | 0.03 | 0.855 |  | -0.02 | -0.04 | 0.01 | 0.142 |  | -0.02 | -0.04 | 0.01 | 0.254 |
|  | **Area** | Urban | * |  |  |  |  | * |  |  |  |  | * |  |  |  |  | * |  |  |  |
|  |  | Rural | 0.00 | -0.02 | 0.03 | 0.640 |  | 0.00 | -0.02 | 0.03 | 0.735 |  | -0.03 | -0.05 | -0.01 | 0.005 |  | -0.03 | -0.06 | -0.01 | 0.009 |
|  | **Overcrowding** | Living w/ others not crowded | * |  |  |  |  | * |  |  |  |  | * |  |  |  |  | * |  |  |  |
|  |  | Alone | 0.01 | -0.02 | 0.03 | 0.646 |  | 0.04 | 0.00 | 0.07 | 0.033 |  | -0.09 | -0.12 | -0.07 | <0.001 | | 0.06 | 0.03 | 0.08 | <0.001 |
|  |  | Living w/ others overcrowded | 0.03 | 0.00 | 0.07 | 0.041 |  | 0.05 | 0.00 | 0.09 | 0.039 |  | 0.04 | 0.01 | 0.08 | 0.021 |  | 0.05 | 0.01 | 0.09 | 0.024 |
|  | **Keyworker** | No | * |  |  |  |  | * |  |  |  |  | * |  |  |  |  | * |  |  |  |
|  |  | Yes | -0.03 | -0.06 | -0.01 | 0.003 |  | -0.05 | -0.08 | -0.01 | 0.006 |  | 0.01 | -0.01 | 0.04 | 0.319 |  | -0.01 | -0.03 | 0.02 | 0.731 |
|  | **Diagnosed physical condition** | None | * |  |  |  |  | * |  |  |  |  | * |  |  |  |  | * |  |  |  |
|  |  | Yes | -0.01 | -0.03 | 0.01 | 0.488 |  | -0.01 | -0.04 | 0.01 | 0.273 |  | 0.00 | -0.02 | 0.02 | 0.713 |  | 0.03 | 0.00 | 0.05 | 0.025 |
|  | **Diagnosed mental condition** | None |  |  |  |  |  |  |  |  |  |  |  |  |  |  |  |  |  |  |  |
|  |  | Yes | 0.00 | -0.03 | 0.02 | 0.809 |  | 0.02 | -0.01 | 0.06 | 0.209 |  | 0.10 | 0.08 | 0.13 | <0.001 | | 0.08 | 0.05 | 0.11 | <0.001 |
|  | **Personality** | Neuroticism | 0.01 | 0.01 | 0.01 | <0.001 | | 0.00 | 0.00 | 0.00 | 0.951 |  | 0.02 | 0.02 | 0.02 | <0.001 | | 0.03 | 0.03 | 0.03 | <0.001 |
|  |  | Extraversion | 0.01 | 0.01 | 0.01 | <0.001 | | 0.01 | 0.01 | 0.01 | <0.001 | | 0.01 | 0.01 | 0.01 | <0.001 | | 0.01 | 0.01 | 0.01 | <0.001 |
|  |  | Openness | 0.03 | 0.02 | 0.03 | <0.001 | | 0.03 | 0.03 | 0.03 | <0.001 | | 0.00 | 0.00 | 0.01 | 0.072 |  | 0.02 | 0.02 | 0.02 | <0.001 |
|  |  | Agreeableness | 0.00 | 0.00 | 0.01 | 0.137 |  | 0.01 | 0.01 | 0.02 | <0.001 | | 0.00 | -0.01 | 0.00 | 0.021 |  | 0.00 | 0.00 | 0.00 | 0.461 |
|  |  | Conscientiousness | 0.01 | 0.01 | 0.02 | <0.001 | | 0.00 | 0.00 | 0.00 | 0.914 |  | -0.01 | -0.01 | 0.00 | <0.001 | | -0.01 | -0.01 | -0.01 | <0.001 |
|  | **Loneliness** | Average loneliness score | 0.04 | 0.03 | 0.04 | <0.001 | | 0.02 | 0.01 | 0.02 | <0.001 | | 0.09 | 0.08 | 0.09 | <0.001 | | 0.06 | 0.06 | 0.07 | <0.001 |
|  | **Social support** | Social support score | 0.01 | 0.01 | 0.01 | <0.001 | | 0.02 | 0.02 | 0.02 | <0.001 | | -0.01 | -0.01 | 0.00 | <0.001 | | 0.04 | 0.04 | 0.04 | <0.001 |

*Displays Model 2 predictors; star indicates reference group*

*fit statistics: problem focused: R^2^= 0.118, AIC=* *1.316; emotion-focused: R^2^= 0.131, AIC= 1.829; avoidant: R^2^=* *0.200, AIC= 1.292; socially supported: R^2^= 0.291, AIC= 1.708*

Table S4 Model 3: Associations of sociodemographic, psychosocial characteristics and adverse events with coping styles

|  |  |  | **Coping 1: Problem-focused** | | | |  | **Coping 2: Emotion-focused** | | | |  | **Coping 3: Avoidant** | | | |  | **Coping 4: Socially supported** | | | |
| --- | --- | --- | --- | --- | --- | --- | --- | --- | --- | --- | --- | --- | --- | --- | --- | --- | --- | --- | --- | --- | --- |
|  |  |  | *Coef* | *95% CI* | | *P* |  | *Coef* | *95% CI* | | *P* |  | *Coef* | *95% CI* | | *P* |  | *Coef* | *95% CI* | | *P* |
|  | **Gender** | Male | * |  |  |  |  | * |  |  |  |  | * |  |  |  |  | * |  |  |  |
|  |  | Female | 0.04 | 0.02 | 0.06 | <0.001 |  | 0.15 | 0.13 | 0.18 | <0.001 |  | 0.03 | 0.01 | 0.05 | 0.002 |  | 0.15 | 0.12 | 0.17 | <0.001 |
|  | **Age group** | 18-29 | * |  |  |  |  | * |  |  |  |  | * |  |  |  |  | * |  |  |  |
|  |  | 30-59 | 0.06 | 0.02 | 0.09 | 0.004 |  | 0.02 | -0.03 | 0.08 | 0.338 |  | -0.02 | -0.06 | 0.02 | 0.409 |  | -0.09 | -0.14 | -0.05 | <0.001 |
|  |  | 60+ | 0.07 | 0.03 | 0.12 | 0.002 |  | 0.04 | -0.02 | 0.10 | 0.215 |  | 0.02 | -0.03 | 0.06 | 0.515 |  | -0.10 | -0.15 | -0.04 | 0.001 |
|  | **Ethnicity** | White | * |  |  |  |  | * |  |  |  |  | * |  |  |  |  | * |  |  |  |
|  |  | BAME | 0.02 | -0.03 | 0.07 | 0.405 |  | 0.06 | -0.01 | 0.12 | 0.076 |  | -0.03 | -0.07 | 0.02 | 0.224 |  | -0.05 | -0.10 | 0.01 | 0.079 |
|  | **Education** | Highest qual/GCSE lower | * |  |  |  |  | * |  |  |  |  | * |  |  |  |  | * |  |  |  |
|  |  | A levels or vocational training | 0.04 | 0.01 | 0.07 | 0.003 |  | 0.08 | 0.04 | 0.11 | <0.001 |  | -0.03 | -0.05 | 0.00 | 0.037 |  | 0.07 | 0.04 | 0.10 | <0.001 |
|  |  | Undergraduate degree | 0.09 | 0.07 | 0.12 | <0.001 |  | 0.12 | 0.08 | 0.15 | <0.001 |  | -0.02 | -0.04 | 0.01 | 0.155 |  | 0.19 | 0.16 | 0.22 | <0.001 |
|  |  | Postgraduate degree | 0.15 | 0.12 | 0.18 | <0.001 |  | 0.14 | 0.10 | 0.17 | <0.001 |  | -0.03 | -0.05 | 0.00 | 0.043 |  | 0.29 | 0.26 | 0.33 | <0.001 |
|  | **Employment** | Employed | * |  |  |  |  | * |  |  |  |  | * |  |  |  |  | * |  |  |  |
|  |  | Student | 0.05 | 0.00 | 0.11 | 0.053 |  | 0.02 | -0.05 | 0.09 | 0.615 |  | 0.03 | -0.04 | 0.11 | 0.346 |  | 0.03 | -0.05 | 0.11 | 0.446 |
|  |  | Inactive | -0.01 | -0.04 | 0.02 | 0.501 |  | -0.05 | -0.08 | -0.01 | 0.015 |  | 0.03 | 0.00 | 0.05 | 0.043 |  | 0.01 | -0.03 | 0.04 | 0.729 |
|  |  | Unemployed | 0.08 | 0.01 | 0.14 | 0.027 |  | -0.01 | -0.09 | 0.07 | 0.742 |  | -0.04 | -0.11 | 0.03 | 0.286 |  | -0.01 | -0.07 | 0.06 | 0.800 |
|  | **Ownership** | Owned | * |  |  |  |  | * |  |  |  |  | * |  |  |  |  | * |  |  |  |
|  |  | Rented/other | -0.03 | -0.05 | 0.00 | 0.024 |  | 0.02 | -0.01 | 0.05 | 0.175 |  | 0.00 | -0.03 | 0.02 | 0.715 |  | 0.04 | 0.01 | 0.07 | 0.004 |
|  | **Low income** | No | * |  |  |  |  | * |  |  |  |  | * |  |  |  |  | * |  |  |  |
|  |  | Yes | 0.00 | -0.02 | 0.02 | 0.943 |  | 0.00 | -0.03 | 0.03 | 0.901 |  | -0.02 | -0.04 | 0.00 | 0.044 |  | -0.02 | -0.04 | 0.01 | 0.210 |
|  | **Area** | Urban | * |  |  |  |  | * |  |  |  |  | * |  |  |  |  | * |  |  |  |
|  |  | Rural | 0.01 | -0.01 | 0.03 | 0.517 |  | 0.01 | -0.02 | 0.03 | 0.634 |  | -0.03 | -0.05 | -0.01 | 0.004 |  | -0.03 | -0.05 | -0.01 | 0.014 |
|  | **Overcrowding** | Living w/ others not crowded | * |  |  |  |  | * |  |  |  |  | * |  |  |  |  | * |  |  |  |
|  |  | Alone | 0.02 | 0.00 | 0.05 | 0.075 |  | 0.04 | 0.01 | 0.08 | 0.007 |  | -0.08 | -0.10 | -0.05 | 0.000 |  | 0.07 | 0.04 | 0.09 | 0.000 |
|  |  | Living w/ others overcrowded | 0.03 | 0.00 | 0.06 | 0.049 |  | 0.04 | 0.00 | 0.09 | 0.044 |  | 0.04 | 0.00 | 0.08 | 0.030 |  | 0.05 | 0.01 | 0.09 | 0.023 |
|  | **Keyworker** | No | * |  |  |  |  | * |  |  |  |  | * |  |  |  |  | * |  |  |  |
|  |  | Yes | -0.03 | -0.05 | -0.01 | 0.015 |  | -0.04 | -0.07 | -0.01 | 0.010 |  | 0.02 | -0.01 | 0.04 | 0.124 |  | -0.01 | -0.04 | 0.02 | 0.708 |
|  | **Diagnosed physical condition** | None | * |  |  |  |  | * |  |  |  |  | * |  |  |  |  | * |  |  |  |
|  |  | Yes | -0.02 | -0.04 | 0.00 | 0.027 |  | -0.02 | -0.05 | 0.00 | 0.072 |  | -0.01 | -0.02 | 0.01 | 0.521 |  | 0.01 | -0.01 | 0.04 | 0.257 |
|  | **Diagnosed mental condition** | None | * |  |  |  |  | * |  |  |  |  | * |  |  |  |  | * |  |  |  |
|  |  | Yes | -0.01 | -0.03 | 0.02 | 0.532 |  | 0.02 | -0.01 | 0.05 | 0.224 |  | 0.09 | 0.06 | 0.12 | <0.001 |  | 0.08 | 0.04 | 0.11 | <0.001 |
|  | **Personality** | Neuroticism | 0.01 | 0.00 | 0.01 | <0.001 |  | 0.00 | -0.01 | 0.00 | 0.106 |  | 0.02 | 0.01 | 0.02 | <0.001 |  | 0.03 | 0.02 | 0.03 | <0.001 |
|  |  | Extraversion | 0.01 | 0.00 | 0.01 | <0.001 |  | 0.01 | 0.01 | 0.01 | <0.001 |  | 0.01 | 0.00 | 0.01 | <0.001 |  | 0.01 | 0.01 | 0.01 | <0.001 |
|  |  | Openness | 0.02 | 0.02 | 0.03 | <0.001 |  | 0.03 | 0.02 | 0.03 | <0.001 |  | 0.00 | 0.00 | 0.00 | 0.604 |  | 0.02 | 0.01 | 0.02 | <0.001 |
|  |  | Agreeableness | 0.00 | 0.00 | 0.01 | 0.160 |  | 0.01 | 0.01 | 0.02 | <0.001 |  | 0.00 | -0.01 | 0.00 | 0.031 |  | 0.00 | 0.00 | 0.01 | 0.404 |
|  |  | Conscientiousness | 0.01 | 0.01 | 0.02 | <0.001 |  | 0.00 | 0.00 | 0.00 | 0.907 |  | -0.01 | -0.01 | 0.00 | <0.001 |  | -0.01 | -0.01 | -0.01 | <0.001 |
|  | **Loneliness** | Average loneliness score | 0.03 | 0.02 | 0.03 | <0.001 |  | 0.01 | 0.00 | 0.02 | 0.002 |  | 0.08 | 0.07 | 0.09 | <0.001 |  | 0.05 | 0.05 | 0.06 | <0.001 |
|  | **Social support** | Social support score | 0.01 | 0.01 | 0.01 | <0.001 |  | 0.02 | 0.02 | 0.02 | <0.001 |  | 0.00 | -0.01 | 0.00 | <0.001 |  | 0.04 | 0.04 | 0.04 | <0.001 |
|  | **Ever had Covid-19** | No | * |  |  |  |  | * |  |  |  |  | * |  |  |  |  | * |  |  |  |
|  |  | Yes | 0.04 | 0.01 | 0.07 | 0.006 |  | 0.06 | 0.02 | 0.09 | 0.003 |  | 0.03 | 0.00 | 0.06 | 0.068 |  | 0.06 | 0.03 | 0.10 | <0.001 |
|  | **Adverse events: finances** | No | * |  |  |  |  | * |  |  |  |  | * |  |  |  |  | * |  |  |  |
|  |  | Yes | 0.08 | 0.05 | 0.10 | <0.001 |  | 0.04 | 0.01 | 0.08 | 0.016 |  | 0.07 | 0.04 | 0.10 | <0.001 |  | 0.01 | -0.03 | 0.04 | 0.671 |
|  | **Adverse events: basic needs** | No | * |  |  |  |  | * |  |  |  |  | * |  |  |  |  | * |  |  |  |
|  |  | Yes | 0.02 | -0.04 | 0.09 | 0.495 |  | 0.00 | -0.10 | 0.09 | 0.951 |  | 0.06 | -0.03 | 0.15 | 0.170 |  | 0.02 | -0.07 | 0.10 | 0.680 |
|  | **Adverse events: Covid-19** | No | * |  |  |  |  | * |  |  |  |  | * |  |  |  |  | * |  |  |  |
|  |  | Yes | -0.06 | -0.16 | 0.03 | 0.204 |  | -0.07 | -0.21 | 0.06 | 0.297 |  | 0.06 | -0.07 | 0.18 | 0.378 |  | -0.15 | -0.26 | -0.04 | 0.008 |
|  | **Worries: finances** | No | * |  |  |  |  | * |  |  |  |  | * |  |  |  |  | * |  |  |  |
|  |  | Yes | 0.09 | 0.07 | 0.11 | <0.001 |  | 0.04 | 0.01 | 0.07 | 0.005 |  | 0.08 | 0.06 | 0.10 | <0.001 |  | 0.10 | 0.07 | 0.13 | <0.001 |
|  | **Worries: basic needs** | No | * |  |  |  |  | * |  |  |  |  | * |  |  |  |  | * |  |  |  |
|  |  | Yes | 0.05 | 0.03 | 0.08 | <0.001 |  | 0.02 | -0.01 | 0.05 | 0.257 |  | 0.06 | 0.04 | 0.09 | <0.001 |  | 0.11 | 0.08 | 0.14 | <0.001 |
|  | **Worries Covid-19** | No | * |  |  |  |  | * |  |  |  |  | * |  |  |  |  | * |  |  |  |
|  |  | Yes | 0.11 | 0.09 | 0.13 | <0.001 |  | 0.09 | 0.07 | 0.12 | <0.001 |  | 0.02 | 0.00 | 0.04 | 0.029 |  | 0.08 | 0.05 | 0.10 | <0.001 |

*Displays Model 3 predictors; star indicates reference group*

*fit statistics: problem focused: R^2^= 0.142, AIC= 1.289; emotion-focused: R^2^= 0.135, AIC=* *1.826; avoidant: R^2^= 0.215, AIC= 1.275; socially supported: R^2^= 0.309, AIC= 1.683*

Supplementary Table S5: Model 1: Unweighted associations of sociodemographic factors with coping styles

|  |  | **Coping 1: Problem-focused** | | | |  | **Coping 2: Emotion-focused** | | | |  | **Coping 3: Avoidant** | | | |  | **Coping 4: Socially supported** | | | |
| --- | --- | --- | --- | --- | --- | --- | --- | --- | --- | --- | --- | --- | --- | --- | --- | --- | --- | --- | --- | --- |
|  |  | *Coef* | *95% CI* | | *P* |  | *Coef* | *95% CI* | | *P* |  | *Coef* | *95% CI* | | *P* |  | *Coef* | *95% CI* | | *P* |
| **Gender** | Male | * |  |  |  |  | * |  |  |  |  | * |  |  |  |  | * |  |  |  |
|  | Female | 0.12 | 0.11 | 0.14 | <0.001 |  | 0.23 | 0.21 | 0.25 | <0.001 |  | 0.09 | 0.08 | 0.11 | <0.001 |  | 0.27 | 0.25 | 0.29 | <0.001 |
| **Age group** | 18-29 | * |  |  |  |  | * |  |  |  |  | * |  |  |  |  | * |  |  |  |
|  | 30-59 | 0.05 | 0.02 | 0.08 | <0.001 |  | 0.03 | 0.00 | 0.07 | 0.079 |  | -0.05 | -0.08 | -0.02 | <0.001 |  | -0.20 | -0.24 | -0.17 | <0.001 |
|  | 60+ | 0.07 | 0.04 | 0.10 | <0.001 |  | 0.06 | 0.02 | 0.10 | 0.006 |  | -0.13 | -0.16 | -0.10 | <0.001 |  | -0.30 | -0.34 | -0.26 | <0.001 |
| **Ethnicity** | White | * |  |  |  |  | * |  |  |  |  | * |  |  |  |  | * |  |  |  |
|  | BAME | 0.04 | 0.01 | 0.07 | 0.016 |  | 0.05 | 0.01 | 0.09 | 0.017 |  | 0.01 | -0.02 | 0.04 | 0.655 |  | -0.04 | -0.08 | 0.00 | 0.058 |
| **Education** | Highest qual/GCSE lower | * |  |  |  |  | * |  |  |  |  | * |  |  |  |  | * |  |  |  |
|  | A levels or vocational training | 0.06 | 0.04 | 0.09 | <0.001 |  | 0.12 | 0.09 | 0.14 | <0.001 |  | -0.02 | -0.04 | 0.01 | 0.187 |  | 0.09 | 0.06 | 0.11 | <0.001 |
|  | Undergraduate degree | 0.14 | 0.12 | 0.16 | <0.001 |  | 0.19 | 0.16 | 0.21 | <0.001 |  | -0.02 | -0.04 | 0.00 | 0.078 |  | 0.21 | 0.19 | 0.24 | <0.001 |
|  | Postgraduate degree | 0.21 | 0.19 | 0.24 | <0.001 |  | 0.24 | 0.21 | 0.26 | <0.001 |  | -0.03 | -0.05 | -0.01 | 0.003 |  | 0.33 | 0.30 | 0.36 | <0.001 |
| **Employment** | Employed | * |  |  |  |  | * |  |  |  |  | * |  |  |  |  | * |  |  |  |
|  | Student | 0.07 | 0.03 | 0.11 | 0.001 |  | 0.00 | -0.05 | 0.05 | 0.996 |  | 0.06 | 0.02 | 0.10 | 0.003 |  | 0.10 | 0.05 | 0.15 | <0.001 |
|  | Inactive | -0.06 | -0.07 | -0.04 | <0.001 |  | -0.07 | -0.09 | -0.05 | <0.001 |  | 0.00 | -0.01 | 0.02 | 0.726 |  | -0.03 | -0.05 | 0.00 | 0.018 |
|  | Unemployed | 0.10 | 0.05 | 0.14 | <0.001 |  | -0.05 | -0.11 | 0.01 | 0.108 |  | 0.05 | 0.01 | 0.10 | 0.021 |  | -0.01 | -0.07 | 0.05 | 0.826 |
| **Ownership** | Owned | * |  |  |  |  | * |  |  |  |  | * |  |  |  |  | * |  |  |  |
|  | Rented/other | 0.01 | -0.01 | 0.02 | 0.400 |  | 0.00 | -0.02 | 0.02 | 0.934 |  | 0.08 | 0.06 | 0.10 | <0.001 |  | 0.05 | 0.03 | 0.07 | <0.001 |
| **Low income** | No | * |  |  |  |  | * |  |  |  |  | * |  |  |  |  | * |  |  |  |
|  | Yes | -0.01 | -0.02 | 0.01 | 0.300 |  | -0.03 | -0.05 | -0.02 | <0.001 |  | 0.05 | 0.04 | 0.06 | <0.001 |  | -0.09 | -0.10 | -0.07 | <0.001 |

*Displays Model 1 predictors; star indicates reference group*

Supplementary Table S6: Model 2: Unweighted associations of psychosocial characteristics with coping styles

|  |  | **Coping 1: Problem-focused** | | | |  | **Coping 2: Emotion-focused** | | | |  | **Coping 3: Avoidant** | | | |  | **Coping 4: Socially supported** | | | |
| --- | --- | --- | --- | --- | --- | --- | --- | --- | --- | --- | --- | --- | --- | --- | --- | --- | --- | --- | --- | --- |
|  |  | *Coef* | *95% CI* | | *P* |  | *Coef* | *95% CI* | | *P* |  | *Coef* | *95% CI* | | *P* |  | *Coef* | *95% CI* | | *P* |
| **Gender** | Male | * |  |  |  |  | * |  |  |  |  | * |  |  |  |  | * |  |  |  |
|  | Female | 0.06 | 0.05 | 0.08 | <0.001 |  | 0.18 | 0.17 | 0.20 | <0.001 |  | 0.04 | 0.03 | 0.05 | <0.001 |  | 0.15 | 0.13 | 0.17 | <0.001 |
| **Age group** | 18-29 | * |  |  |  |  | * |  |  |  |  | * |  |  |  |  | * |  |  |  |
|  | 30-59 | 0.08 | 0.05 | 0.10 | <0.001 |  | 0.06 | 0.02 | 0.09 | 0.001 |  | 0.00 | -0.03 | 0.02 | 0.741 |  | -0.07 | -0.11 | -0.04 | <0.001 |
|  | 60+ | 0.10 | 0.07 | 0.13 | <0.001 |  | 0.06 | 0.02 | 0.10 | 0.004 |  | 0.01 | -0.02 | 0.04 | 0.664 |  | -0.09 | -0.13 | -0.06 | <0.001 |
| **Ethnicity** | White | * |  |  |  |  | * |  |  |  |  | * |  |  |  |  | * |  |  |  |
|  | BAME | 0.04 | 0.01 | 0.06 | 0.018 |  | 0.05 | 0.01 | 0.09 | 0.013 |  | 0.01 | -0.02 | 0.04 | 0.637 |  | -0.02 | -0.06 | 0.01 | 0.244 |
| **Education** | Highest qual/GCSE lower | * |  |  |  |  | * |  |  |  |  | * |  |  |  |  | * |  |  |  |
|  | A levels or vocational training | 0.04 | 0.02 | 0.06 | <0.001 |  | 0.09 | 0.06 | 0.12 | <0.001 |  | -0.02 | -0.04 | 0.00 | 0.062 |  | 0.07 | 0.05 | 0.10 | <0.001 |
|  | Undergraduate degree | 0.10 | 0.08 | 0.12 | <0.001 |  | 0.13 | 0.11 | 0.16 | <0.001 |  | -0.01 | -0.03 | 0.00 | 0.132 |  | 0.18 | 0.16 | 0.21 | <0.001 |
|  | Postgraduate degree | 0.16 | 0.14 | 0.18 | <0.001 |  | 0.16 | 0.13 | 0.18 | <0.001 |  | -0.02 | -0.04 | 0.00 | 0.022 |  | 0.28 | 0.26 | 0.31 | <0.001 |
| **Employment** | Employed | * |  |  |  |  | * |  |  |  |  | * |  |  |  |  | * |  |  |  |
|  | Student | 0.05 | 0.01 | 0.08 | 0.019 |  | -0.02 | -0.07 | 0.03 | 0.391 |  | 0.02 | -0.02 | 0.06 | 0.331 |  | 0.05 | 0.00 | 0.09 | 0.053 |
|  | Inactive | -0.05 | -0.06 | -0.03 | <0.001 |  | -0.06 | -0.08 | -0.04 | <0.001 |  | -0.01 | -0.03 | 0.00 | 0.085 |  | -0.04 | -0.06 | -0.02 | <0.001 |
|  | Unemployed | 0.09 | 0.05 | 0.13 | <0.001 |  | -0.03 | -0.09 | 0.02 | 0.225 |  | 0.00 | -0.04 | 0.04 | 0.931 |  | 0.00 | -0.05 | 0.06 | 0.882 |
| **Ownership** | Owned | * |  |  |  |  | * |  |  |  |  | * |  |  |  |  | * |  |  |  |
|  | Rented/other | -0.01 | -0.02 | 0.01 | 0.262 |  | 0.02 | 0.00 | 0.04 | 0.102 |  | 0.01 | -0.01 | 0.02 | 0.496 |  | 0.04 | 0.03 | 0.06 | <0.001 |
| **Low income** | No | * |  |  |  |  | * |  |  |  |  | * |  |  |  |  | * |  |  |  |
|  | Yes | 0.00 | -0.01 | 0.02 | 0.766 |  | 0.01 | 0.00 | 0.03 | 0.106 |  | -0.01 | -0.02 | 0.01 | 0.270 |  | -0.02 | -0.04 | -0.01 | 0.005 |
| **Area** | Urban | * |  |  |  |  | * |  |  |  |  | * |  |  |  |  | * |  |  |  |
|  | Rural | 0.01 | 0.00 | 0.02 | 0.115 |  | 0.00 | -0.02 | 0.02 | 0.950 |  | -0.01 | -0.02 | 0.00 | 0.174 |  | -0.04 | -0.06 | -0.02 | <0.001 |
| **Overcrowding** | Living w/ others not crowded | * |  |  |  |  | * |  |  |  |  | * |  |  |  |  | * |  |  |  |
|  | Alone | 0.02 | 0.00 | 0.03 | 0.061 |  | 0.06 | 0.04 | 0.08 | <0.001 |  | -0.09 | -0.10 | -0.07 | <0.001 |  | 0.09 | 0.07 | 0.11 | <0.001 |
|  | Living w/ others overcrowded | 0.06 | 0.04 | 0.08 | <0.001 |  | 0.05 | 0.02 | 0.08 | 0.001 |  | 0.03 | 0.00 | 0.05 | 0.017 |  | 0.06 | 0.03 | 0.08 | <0.001 |
| **Keyworker** | No | * |  |  |  |  | * |  |  |  |  | * |  |  |  |  | * |  |  |  |
|  | Yes | 0.00 | -0.01 | 0.02 | 0.869 |  | -0.01 | -0.03 | 0.01 | 0.326 |  | 0.01 | 0.00 | 0.03 | 0.104 |  | 0.01 | -0.01 | 0.02 | 0.521 |
| **Diagnosed physical condition** | None | * |  |  |  |  | * |  |  |  |  | * |  |  |  |  | * |  |  |  |
|  | Yes | 0.01 | -0.01 | 0.02 | 0.417 |  | -0.01 | -0.03 | 0.00 | 0.106 |  | -0.01 | -0.02 | 0.01 | 0.254 |  | 0.01 | 0.00 | 0.03 | 0.085 |
| **Diagnosed mental condition** | None | * |  |  |  |  | * |  |  |  |  | * |  |  |  |  | * |  |  |  |
|  | Yes | -0.02 | -0.03 | 0.00 | 0.063 |  | 0.02 | 0.00 | 0.04 | 0.126 |  | 0.10 | 0.09 | 0.12 | <0.001 |  | 0.09 | 0.07 | 0.11 | <0.001 |
| **Personality** | Neuroticism | 0.01 | 0.01 | 0.01 | <0.001 |  | 0.00 | 0.00 | 0.00 | 0.005 |  | 0.02 | 0.02 | 0.02 | <0.001 |  | 0.03 | 0.03 | 0.03 | <0.001 |
|  | Extraversion | 0.01 | 0.01 | 0.01 | <0.001 |  | 0.01 | 0.01 | 0.01 | <0.001 |  | 0.01 | 0.01 | 0.01 | <0.001 |  | 0.01 | 0.01 | 0.01 | <0.001 |
|  | Openness | 0.03 | 0.02 | 0.03 | <0.001 |  | 0.03 | 0.03 | 0.03 | <0.001 |  | 0.00 | 0.00 | 0.00 | 0.007 |  | 0.02 | 0.02 | 0.02 | <0.001 |
|  | Agreeableness | 0.00 | 0.00 | 0.01 | <0.001 |  | 0.02 | 0.01 | 0.02 | <0.001 |  | 0.00 | -0.01 | 0.00 | <0.001 |  | 0.00 | 0.00 | 0.00 | 0.066 |
|  | Conscientiousness | 0.01 | 0.01 | 0.02 | <0.001 |  | 0.00 | 0.00 | 0.00 | 0.564 |  | -0.01 | -0.01 | -0.01 | <0.001 |  | -0.01 | -0.01 | -0.01 | <0.001 |
| **Loneliness** | Average loneliness score | 0.03 | 0.03 | 0.04 | <0.001 |  | 0.00 | 0.00 | 0.01 | 0.446 |  | 0.08 | 0.08 | 0.09 | <0.001 |  | 0.06 | 0.06 | 0.07 | <0.001 |
| **Social support** | Social support score | 0.01 | 0.01 | 0.01 | <0.001 |  | 0.02 | 0.02 | 0.02 | <0.001 |  | 0.00 | -0.01 | 0.00 | <0.001 |  | 0.04 | 0.04 | 0.04 | <0.001 |

*Displays Model 2 predictors; star indicates reference group*

Supplementary Table S7: Model 3: Unweighted associations of worries and adverse events with coping styles

|  |  | **Coping 1: Problem-focused** | | | |  | **Coping 2: Emotion-focused** | | | |  | **Coping 3: Avoidant** | | | |  | **Coping 4: Socially supported** | | | |
| --- | --- | --- | --- | --- | --- | --- | --- | --- | --- | --- | --- | --- | --- | --- | --- | --- | --- | --- | --- | --- |
|  |  | *Coef* | *95% CI* | | *P* |  | *Coef* | *95% CI* | | *P* |  | *Coef* | *95% CI* | | *P* |  | *Coef* | *95% CI* | | *P* |
| **Gender** | Male | * |  |  |  |  | * |  |  |  |  | * |  |  |  |  | * |  |  |  |
|  | Female | 0.06 | 0.05 | 0.07 | <0.001 |  | 0.06 | 0.05 | 0.07 | <0.001 |  | 0.04 | 0.03 | 0.05 | <0.001 |  | 0.15 | 0.13 | 0.16 | <0.001 |
| **Age group** | 18-29 | * |  |  |  |  | * |  |  |  |  | * |  |  |  |  | * |  |  |  |
|  | 30-59 | 0.06 | 0.04 | 0.09 | <0.001 |  | 0.06 | 0.04 | 0.09 | <0.001 |  | -0.01 | -0.04 | 0.02 | 0.426 |  | -0.08 | -0.11 | -0.05 | <0.001 |
|  | 60+ | 0.10 | 0.07 | 0.13 | <0.001 |  | 0.10 | 0.07 | 0.13 | <0.001 |  | 0.02 | -0.01 | 0.05 | 0.211 |  | -0.08 | -0.12 | -0.05 | <0.001 |
| **Ethnicity** | White | * |  |  |  |  | * |  |  |  |  | * |  |  |  |  | * |  |  |  |
|  | BAME | 0.02 | 0.00 | 0.05 | 0.097 |  | 0.02 | 0.00 | 0.05 | 0.097 |  | 0.00 | -0.03 | 0.03 | 0.924 |  | -0.03 | -0.07 | 0.00 | 0.076 |
| **Education** | Highest qual/GCSE lower | * |  |  |  |  | * |  |  |  |  | * |  |  |  |  | * |  |  |  |
|  | A levels or vocational training | 0.04 | 0.02 | 0.06 | <0.001 |  | 0.04 | 0.02 | 0.06 | <0.001 |  | -0.02 | -0.04 | 0.00 | 0.022 |  | 0.07 | 0.04 | 0.09 | <0.001 |
|  | Undergraduate degree | 0.10 | 0.08 | 0.12 | <0.001 |  | 0.10 | 0.08 | 0.12 | <0.001 |  | -0.02 | -0.04 | 0.00 | 0.044 |  | 0.17 | 0.15 | 0.20 | <0.001 |
|  | Postgraduate degree | 0.15 | 0.13 | 0.17 | <0.001 |  | 0.15 | 0.13 | 0.17 | <0.001 |  | -0.03 | -0.05 | -0.01 | 0.004 |  | 0.27 | 0.24 | 0.29 | <0.001 |
| **Employment** | Employed | * |  |  |  |  | * |  |  |  |  | * |  |  |  |  | * |  |  |  |
|  | Student | 0.06 | 0.03 | 0.10 | 0.001 |  | 0.06 | 0.03 | 0.10 | 0.001 |  | 0.04 | 0.00 | 0.07 | 0.065 |  | 0.06 | 0.02 | 0.11 | 0.008 |
|  | Inactive | -0.01 | -0.03 | 0.01 | 0.170 |  | -0.01 | -0.03 | 0.01 | 0.170 |  | 0.02 | 0.00 | 0.03 | 0.073 |  | 0.00 | -0.02 | 0.02 | 0.977 |
|  | Unemployed | 0.09 | 0.05 | 0.13 | <0.001 |  | 0.09 | 0.05 | 0.13 | <0.001 |  | 0.00 | -0.04 | 0.04 | 0.966 |  | 0.01 | -0.04 | 0.07 | 0.606 |
| **Ownership** | Owned | * |  |  |  |  | * |  |  |  |  | * |  |  |  |  | * |  |  |  |
|  | Rented/other | -0.01 | -0.03 | 0.00 | 0.057 |  | -0.01 | -0.03 | 0.00 | 0.057 |  | 0.00 | -0.02 | 0.01 | 0.683 |  | 0.04 | 0.02 | 0.06 | <0.001 |
| **Low income** | No | * |  |  |  |  | * |  |  |  |  | * |  |  |  |  | * |  |  |  |
|  | Yes | 0.00 | -0.02 | 0.01 | 0.477 |  | 0.00 | -0.02 | 0.01 | 0.477 |  | -0.02 | -0.03 | 0.00 | 0.028 |  | -0.03 | -0.04 | -0.01 | 0.001 |
| **Area** | Urban | * |  |  |  |  | * |  |  |  |  | * |  |  |  |  | * |  |  |  |
|  | Rural | 0.01 | 0.00 | 0.03 | 0.063 |  | 0.01 | 0.00 | 0.03 | 0.063 |  | -0.01 | -0.02 | 0.00 | 0.198 |  | -0.04 | -0.05 | -0.02 | <0.001 |
| **Overcrowding** | Living w/ others not crowded | * |  |  |  |  | * |  |  |  |  | * |  |  |  |  | * |  |  |  |
|  | Alone | 0.03 | 0.02 | 0.05 | <0.001 |  | 0.03 | 0.02 | 0.05 | <0.001 |  | -0.08 | -0.09 | -0.06 | <0.001 |  | 0.10 | 0.08 | 0.12 | <0.001 |
|  | Living w/ others overcrowded | 0.05 | 0.03 | 0.08 | <0.001 |  | 0.05 | 0.03 | 0.08 | <0.001 |  | 0.02 | 0.00 | 0.04 | 0.041 |  | 0.05 | 0.03 | 0.08 | <0.001 |
| **Keyworker** | No | * |  |  |  |  | * |  |  |  |  | * |  |  |  |  | * |  |  |  |
|  | Yes | 0.00 | -0.01 | 0.02 | 0.549 |  | 0.00 | -0.01 | 0.02 | 0.549 |  | 0.02 | 0.00 | 0.03 | 0.042 |  | 0.00 | -0.01 | 0.02 | 0.703 |
| **Diagnosed physical condition** | None | * |  |  |  |  | * |  |  |  |  | * |  |  |  |  | * |  |  |  |
|  | Yes | -0.01 | -0.02 | 0.00 | 0.105 |  | -0.01 | -0.02 | 0.00 | 0.105 |  | -0.02 | -0.03 | -0.01 | 0.005 |  | 0.00 | -0.02 | 0.01 | 0.683 |
| **Diagnosed mental condition** | None | * |  |  |  |  | * |  |  |  |  | * |  |  |  |  | * |  |  |  |
|  | Yes | -0.02 | -0.04 | 0.00 | 0.011 |  | -0.02 | -0.04 | 0.00 | 0.011 |  | 0.09 | 0.08 | 0.11 | <0.001 |  | 0.08 | 0.06 | 0.10 | <0.001 |
| **Personality** | Neuroticism | 0.01 | 0.00 | 0.01 | <0.001 |  | 0.01 | 0.00 | 0.01 | <0.001 |  | 0.02 | 0.01 | 0.02 | <0.001 |  | 0.02 | 0.02 | 0.02 | <0.001 |
|  | Extraversion | 0.01 | 0.01 | 0.01 | <0.001 |  | 0.01 | 0.01 | 0.01 | <0.001 |  | 0.01 | 0.01 | 0.01 | <0.001 |  | 0.01 | 0.01 | 0.01 | <0.001 |
|  | Openness | 0.02 | 0.02 | 0.03 | <0.001 |  | 0.02 | 0.02 | 0.03 | <0.001 |  | 0.00 | 0.00 | 0.00 | 0.640 |  | 0.02 | 0.02 | 0.02 | <0.001 |
|  | Agreeableness | 0.00 | 0.00 | 0.01 | <0.001 |  | 0.00 | 0.00 | 0.01 | <0.001 |  | 0.00 | -0.01 | 0.00 | <0.001 |  | 0.00 | 0.00 | 0.00 | 0.118 |
|  | Conscientiousness | 0.01 | 0.01 | 0.02 | <0.001 |  | 0.01 | 0.01 | 0.02 | <0.001 |  | -0.01 | -0.01 | -0.01 | <0.001 |  | -0.01 | -0.01 | -0.01 | <0.001 |
| **Loneliness** | Average loneliness score | 0.03 | 0.02 | 0.03 | <0.001 |  | 0.03 | 0.02 | 0.03 | <0.001 |  | 0.08 | 0.07 | 0.08 | <0.001 |  | 0.05 | 0.05 | 0.06 | <0.001 |
| **Social support** | Social support score | 0.01 | 0.01 | 0.01 | <0.001 |  | 0.01 | 0.01 | 0.01 | <0.001 |  | 0.00 | 0.00 | 0.00 | <0.001 |  | 0.04 | 0.04 | 0.04 | <0.001 |
| **Ever had Covid-19** | No | * |  |  |  |  | * |  |  |  |  | * |  |  |  |  | * |  |  |  |
|  | Yes | 0.04 | 0.03 | 0.06 | <0.001 |  | 0.04 | 0.03 | 0.06 | <0.001 |  | 0.03 | 0.01 | 0.05 | 0.001 |  | 0.05 | 0.03 | 0.08 | <0.001 |
| **Adverse events: finances** | No | * |  |  |  |  | * |  |  |  |  | * |  |  |  |  | * |  |  |  |
|  | Yes | 0.07 | 0.05 | 0.08 | <0.001 |  | 0.07 | 0.05 | 0.08 | <0.001 |  | 0.05 | 0.03 | 0.06 | <0.001 |  | -0.01 | -0.03 | 0.01 | 0.234 |
| **Adverse events: basic needs** | No | * |  |  |  |  | * |  |  |  |  | * |  |  |  |  | * |  |  |  |
|  | Yes | 0.02 | -0.03 | 0.07 | 0.475 |  | 0.02 | -0.03 | 0.07 | 0.475 |  | 0.04 | 0.00 | 0.09 | 0.070 |  | -0.01 | -0.06 | 0.05 | 0.841 |
| **Adverse events: Covid-19** | No | * |  |  |  |  | * |  |  |  |  | * |  |  |  |  | * |  |  |  |
|  | Yes | -0.05 | -0.11 | 0.01 | 0.100 |  | -0.05 | -0.11 | 0.01 | 0.100 |  | 0.06 | 0.00 | 0.13 | 0.047 |  | -0.10 | -0.17 | -0.02 | 0.014 |
| **Worries: finances** | No | * |  |  |  |  | * |  |  |  |  | * |  |  |  |  | * |  |  |  |
|  | Yes | 0.08 | 0.07 | 0.10 | <0.001 |  | 0.08 | 0.07 | 0.10 | <0.001 |  | 0.08 | 0.07 | 0.09 | <0.001 |  | 0.12 | 0.10 | 0.14 | <0.001 |
| **Worries: basic needs** | No | * |  |  |  |  | * |  |  |  |  | * |  |  |  |  | * |  |  |  |
|  | Yes | 0.06 | 0.05 | 0.07 | <0.001 |  | 0.06 | 0.05 | 0.07 | <0.001 |  | 0.07 | 0.06 | 0.09 | <0.001 |  | 0.11 | 0.09 | 0.13 | <0.001 |
| **Worries Covid-19** | No | * |  |  |  |  | * |  |  |  |  | * |  |  |  |  | * |  |  |  |
|  | Yes | 0.09 | 0.08 | 0.10 | <0.001 |  | 0.09 | 0.08 | 0.10 | <0.001 |  | 0.02 | 0.01 | 0.03 | <0.001 |  | 0.09 | 0.07 | 0.10 | <0.001 |

*Displays Model 3 predictors; star indicates reference group*
